# Abdominal Tuberculosis in Childhood

**Published:** 1907-11-30

**Authors:** William P. S. Branson

**Affiliations:** Assistant Physician, East London Hospital for Children.


### ABDOMINAL TUBERCULOSIS IN CHILDHOOD.

By WILLIAM P. S. BRANSON, M.D., M.R.C.P., Assistant Physician, East London Hospital for Children.

THERE are three elementary lesions in abdominal tuberculosis, those of the gut, of the mesenteric glands, and of the peritoneum. Any one of these may occur alone for a time, but in practice the only one which is commonly found unaccompanied is that of the peritoneal. In almost every case the peritoneal trouble is most prominent clinically, and [illegible]sequence "tuberculous peritonitis" implies as a rule a combination of two or more of the elementary lesions. Pathologically the lesion of the gut is ulceration; the ulcers attack the ileum for choice, and are transverse to its axis, thus differing from those of typhoid fever. It is usual to find miliary tubercles on the serous surface of these ulcers. The lesion of the mesenteric glands is caseation. There is no doubt that, like the bronchial, so the mesenteric glands may become infected without any discoverable lesion of the mucous membrane; for it is no rarity to find one or two caseous mesenteric glands in the bodies of children who show no signs of tuberculosis of the intestine or peritoneum: but anything like massive enlargement of these glands does as a fact generally connote intestinal ulceration. The lesions of the peritoneum are of two varieties—(1) miliary tuberculosis; (2) adhesive peritonitis. The miliary variety is commonly accompanied by ascites, while the other is usually dry, but combinations of the two are not infrequent. In advanced cases the intestines become inextricably matted together and to the abdominal parietes. In these circumstances abscess-formation may occur. Such an abscess commonly points at or about the umbilicus, leaving a fistula which passes either to a mass of disintegrating caseous glands or, in consequence of destructive ulceration, into the intestinal lumen.

As far as my own experience goes, when the disease appears clinically as a primary tuberculosis there, autopsy shows that in about 50 per cent. of cases the infection is limited to the belly. It seems certain, therefore, that the infection is often food-borne, probably by tuberculous milk.

Miliary tuberculosis of the peritoneum may give no sign at all of its existence, and surgeons not infrequently come upon it accidentally in the course of abdominal operations of convenience, such as that for the radical cure of hernia. More often, however, there is definite ascites, associated with a pallor or loss of vigour. Such ascitic cases are clinically very mild, and often afebrile throughout, but it is necessary to remember that they may develop the graver plastic form.

The latter form of the disease is invariably associated with definite loss of health, and often with definite localising symptoms. The chief of these is pain, referred to the region of the umbilicus, not very severe, but recurrent. Examination of the belly in these cases reveals distension, due commonly to a partial obstruction. This obstruction depends upon interference by adhesions with the free peristalsis of the gut. Occasionally actual kinking of the gut takes place, in which case the symptoms are those of acute intestinal obstruction; and such obstruction may be the first indication of the tuberculous disease, so insidious is the onset at times. The typical swollen belly of tuberculous peritonitis offers a diffuse resistance and suggestion of "doughiness" which is very characteristic on palpation. There is generally no tenderness, and even when doughiness is absent it may be possible to detect local swellings indicative of intestinal matting, of loculated effusions, or of caseous lymphatic masses. In advanced cases one sees a red and pouting navel, perhaps discharging fæcal or watery fluid, and this is pathognomonic of tuberculous peritonitis. Diarrhœa is not the rule until a late period of the disease when ulceration of the gut is extensive, but some irregularity in the action of the bowels is common.

### Diagnosis.

The first two years of life, and particularly the first, are seldom visited by abdominal tuberculosis. Most of the cases of chronic diarrhœa in infancy which may raise a suspicion of tuberculosis should properly be referred to other categories. But there is a disease of older children which bears many features of resemblance to the tuberculous malady. This is what Dr. Eustace Smith calls "Mucous disease," and others "chronic intestinal dyspepsia." It is characterised by loss of weight and health, tympanites, and unhealthy motions, often accompanied by a profuse discharge of mucus from the intestine. Children affected with this condition may present the aspect of tuberculous patients, especially the dry skin and "muddy" complexion. The temperature chart is of great use in diagnosis, for in tuberculous cases of any severity there is almost always an irregular pyrexia, while in mucous disease it is the exception, apart from complications such as bronchitis or bronchopneumonia. Further, although distension of the belly is common to both maladies, in the latter there is evidence neither of ascites nor of intestinal matting, and the belly remains easily palpable. There is seldom any difficulty in distinguishing between abdominal tuberculosis and typhoid fever, and doubt on the subject is easily disposed of by means of Widal's reaction. The rare condition of diffuse sarcomatosis of the peritoneum is almost certain to be labelled tuberculous peritonitis. I know of no means of identifying it, but its rarity renders it almost negligible. Lastly, the possibility of tuberculous peritonitis should always be borne in mind whenever a child under the age of five years presents the symptoms of acute intestinal obstruction.

When tuberculosis of the belly complicates the same disease elsewhere the outlook in children is very grave indeed, and becomes almost desperate when profuse and constant diarrhœa indicates intestinal ulceration. But the case is far otherwise when the disease appears to be local in the belly. In this case it is not too much to say that everything turns upon early diagnosis and a long purse, for treatment to be effectual must be protracted. For this reason the prospect of cure for a hospital patient is infinitely less favourable, *cæteris paribus*, than for the child of well-to-do parents. I have made some inquiry into the fate of these patients at the East London Hospital,* and find that the death-rate lies somewhere between sixty and seventy per cent.; of those who recover a fair proportion present, after some years' interval, persistent local evidences of the past disease. The prognosis for the ascitic variety is distinctly better than for the adhesive form, but even so is not favourable.

### Treatment.

There is a difference of opinion as to the value of simple incision of the belly for the relief of the ascitic cases. My own experience is to the effect that it is unnecessary as a rule, since the effusion usually disappears spontaneously in a few weeks, while the sites of such incisions often become the seat of tuberculous granulations. Generally speaking, the chief item in treatment is that it should be commenced early, before, that is to say, large masses of necrotic caseous material have accumulated in the belly. There is no remedy of particular virtue, and attention should be concentrated on the reinforcing of the constitution. Rest should be taken, as far as the circumstances of the case admit, in the open air; an abundant supply of milk, and close attention to the regular action of the bowels to prevent accumulations of gas or fæces and the discomfort and risks of obstruction attendant upon them are also indicated. So long as there is fever the patient should be kept recumbent, but out of doors all day. Even in a small garden it [illegible] matter of no great difficulty to erect a summer-house to give protection from the weather, and in this the child should spend his days for four or five months. This open-air treatment, however, requires to be tempered by discretion and regard for climatic conditions; and it is better, in my judgment, that a child should lie in a warmed room by the open window rather than be submitted to the rigours of bitter weather in order to conform strictly to the terms of the "cure."

* Med. Chir. Trans. vol. lxxxviii.

